# The Effect of Combined Superheated Steam Roasting and Smoking on the Quality Characteristic of Alaska Pollack (*Gadus chalcogrammus*) Roe

**DOI:** 10.3390/foods10123047

**Published:** 2021-12-08

**Authors:** Gabriel Tirtawijaya, Jin-Hwa Lee, Jong-Su Jang, Do-Youb Kim, Jae-Hak Sohn, Jae-Suk Choi

**Affiliations:** 1 Seafood Research Center, Industry Academy Cooperation Foundation (IACF), Silla University, 606, Advanced Seafood Processing Complex, Amnam-dong, Wonyang-ro, Seo-gu, Busan 49277, Korea; tirtawijayag@yahoo.com (G.T.); evolution_5237@naver.com (J.-H.L.); jhsohn@silla.ac.kr (J.-H.S.); 2 Faculty of Biotechnology, University of Surabaya, Jalan Raya Kalirungkut Surabaya, Surabaya 60292, Indonesia; 3 Deok-Hwa Food Co., Ltd., 3F, Domajang-dong, 35, Wonyang-ro, Seo-gu, Busan 49277, Korea; jsjang@dhfood.kr (J.-S.J.); kim926@dhfood.kr (D.-Y.K.); 4 Department of Food Biotechnology, College of Medical and Life Sciences, Silla University, 140, Baegyang-daero 700 beon-gil, Sasang-gu, Busan 46958, Korea

**Keywords:** Alaska pollack roe, superheated steam roasting, smoking, product quality

## Abstract

Alaska pollack roe (APR) is a protein source that is usually salted and fermented, containing a high salt content. Using a combination of superheated steam roasting and smoking, we developed a new low-salt ready-to-eat APR variant, whose quality characteristics we analyzed. The optimal conditions for roasting (216 °C for 4 min) and smoking (64 °C for 14 min) were obtained from sensorial attributes using response surface methodology. Under the optimal conditions, smoke-roasted APR had an overall acceptance (OA) score of 8.89. The combination of roasting and smoking significantly increased volatile basic nitrogen (VBN, 18.6%) and decreased the total bacterial count (TBC, 38.6%), while thiobarbituric acid reactive substances (TBARS) were not affected. Smoke-roasting APR also increased its nutritional content to 30% protein with 44% essential amino acids, and more than 40% DHA and EPA in 4.3% fat. During 30 days of storage, the OA, VBN, TBARS, and TBC values significantly changed with time and storage temperature (*p* < 0.05). The shelf life of the product was estimated to be 24 d. In conclusion, the combination of roasting and smoking APR could improve product quality and may be an alternative to diversify processed APR.

## 1. Introduction

Fish roes are commercial seafood products that are consumed worldwide. Roes from sturgeon, salmon, herring, mullet, cod, crab, sea urchin, and pollack are commonly consumed [1,2,3]. Fish roes are marketed as fresh chilled or frozen products, which are then processed to extend their shelf life. Processed fish roes are popular culinary products frequently used as ready-to-eat (RTE) side dishes in South Korea. Alaska pollack roe (APR) is one of the most imported roes in South Korea, contributing to nearly half of all imported fish roes, with 15,285 MT of frozen roe imported in 2019, an increase of 3.7% from the previous year [4]. 

APR constitutes 5% of the weight of fish but contributes 31% of the product value [5]. This roe has been used as a food source owing to its good nutritional value. It contains 19.2–22.4% protein, 1.5–2.8% lipids, and 1.1–4.4% ash [6,7,8,9]. Salting and fermentation processes are commonly used to extend the shelf life of APR [10]. Approximately 20–30% salt is used in salting and fermenting seafood [3,11]. Salt-curing and fermentation have become a problem for fish roe processing as market awareness of healthy food has increased. At present, the market prefers to eat foods with low salt contents. Several studies have been conducted on using lower salt concentrations (8–12%) in APR processing, but salt contents are still 3.6–7.0 g of salt per 100 g of final products [12,13]. The World Health Organization (WHO) recommends less than 5 g/day of salt for adults to reduce blood pressure and the risk of cardiovascular disease (CVD) [14]. Consequently, salt intake needs to be controlled, and the formulation of food products contributes to salt intake reduction [15]. 

The combination of superheated steam roasting (SSR) and smoking treatments is a processing technology that can produce superior-quality and longer-shelf life seafood products with acceptable sensory, physicochemical, and microbial properties [16,17,18]. Processing methods that use SSR are known to have the benefit of producing good-quality products while utilizing less energy owing to shorter processing times [19]. In addition, SSR products have been observed to have a desirable texture and appearance [20], as well as aiding in a reduction in food oxidation [21,22]. Moreover, SSR has been utilized in various seafood processing methods, such as roasting, steaming, and braising [17,18,23]. In the current study, APR was processed by a smoking treatment after roasting. Smoking is widely used for preserving fish and is often combined with drying, salting, or heating processes [24]. Smoking has been reported to increase food sensory acceptability, inhibit microbial growth [25,26], improve physicochemical properties, and extend the shelf life of half-dried Pacific saury [27]. However, with longer processing times and higher temperatures, SSR and smoking treatments may generate undesirable sensory characteristics, including product shrinkage, excessive moisture loss, and a charred taste and aroma. Conversely, shorter processing times and lower temperatures may result in an undercooked taste or a microbiologically unsafe product. Therefore, optimizing roast-smoked APR processing conditions are important and could be useful for meeting consumer acceptability.

Here, we developed a new processing method for APR to overcome the high-salt roe problem and increase RTE seafood diversification. We evaluated APR processing using a combination of SSR and smoking. In this study, response surface methodology (RSM) was used to design the experiments and optimize the processing conditions. The RTE APR was developed using optimal processing conditions, and the sensorial, chemical, microbial, and nutritional properties were characterized. The final product was stored for a certain period to assess its shelf life. This characterization is valuable for manufacturers and researchers in new product development. 

## 2. Materials and Methods

### 2.1. Preparation of the Raw APR

Smoke-roasted APR was prepared from seasoned raw APR, obtained from Deok-Hwa Food Co. Ltd., Busan, Korea, in a frozen state. The seasoning process of raw APR began by washing and disinfecting with salt (3%) and sodium hypochlorite (200 ppm) for 1 min. Thereafter, refined salt (0.88%), sorbitol powder (8.8%), sodium _L_-glutamate (0.88%), rice wine (0.88%), fermented lactic acid bacteria (0.5%), and sodium ascorbate (0.3%) were mixed with raw APR (87.8%) at 30 °C. After 6 h, seasoned raw APR was drained, transferred to a clean container, and allowed to stand for 24 h at 5–10 °C. Afterward, the seasoned raw APR was washed and disinfected thrice with salt (3%) and sodium hypochlorite (200 ppm) for 10 s each time before packaging and freezing.

Frozen uncut roe or untorn ovaries of APR (20–30 g) were selected for this study. Frozen APR samples were thawed using three different methods: water thawing (WT, 20–22 °C), air thawing (AT, 18–20 °C), and high-frequency defrosting (HFD; TEMPERTRON FRT-10, Yamamoto Vinita Co. Ltd., Osaka, Japan). The thawing method selected for further processing was based on the percentage drip loss, which was measured by the difference in APR weight before and after the thawing process, divided by the initial weight, and then multiplied by 100%. 

### 2.2. Production of RTE Smoke-Roasted APR

**Figure 1:**
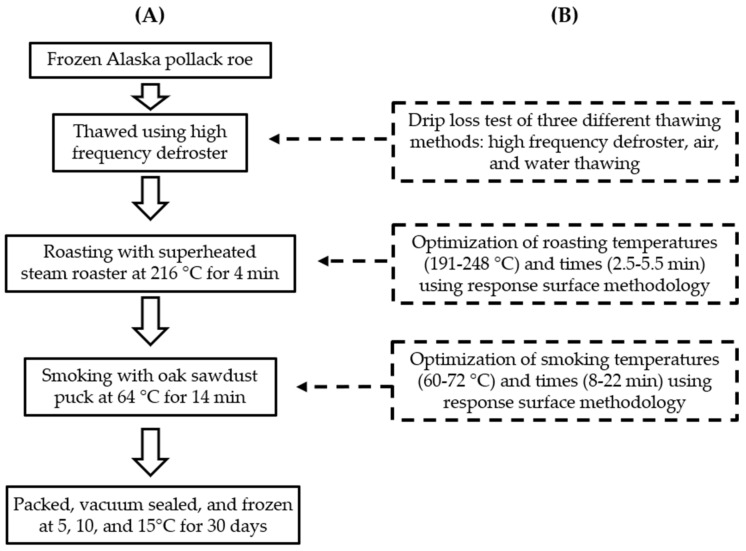
Processing steps of smoke-roasted Alaska pollack roe. The final product processing flow (**A**) was obtained from the designed experiment (**B**).

The RTE smoke-roasted APR was produced through various steps, including roasting APR using SSR, the smoking of roasted APR, the vacuum packaging of smoke-roasted APR, and pasteurization of the final product, as shown in Figure 1. The roasting and smoking processes were conducted by optimizing the processing conditions, as described in the succeeding sections. The optimization of roasting and smoking conditions was based on evaluating sensorial properties to assess market preferences and was performed using the response optimizer function in the Minitab program. The target for sensorial properties was set at 9 as the highest score on the hedonic scale.

The smoke-roasted APR samples were packed in polypropylene plastic bags and vacuum-sealed using a double-chambered vacuum packer (HFV-1180D; Hankook Fujee Machinery Co., Ltd., Hwaseong-si, Korea). For shelf life evaluation, packaged samples were pasteurized at 65 °C for 15 min before storage.

#### 2.2.1. Optimization of SSR Conditions for APR Processing

Thawed APR samples were roasted using an SSR Aero Steam Oven (DFC-560A-2R/L; Naomoto Co., Osaka, Japan) at different roasting temperatures and times as factors. The 11 roasting temperature and time combinations were determined by RSM using a central composite design, as follows: 200 °C for 3 min, 240 °C for 3 min, 200 °C for 5 min, 240 °C for 5 min, 191 °C for 4 min, 248 °C for 4 min, 220 °C for 2.5 min, 220 °C for 5.5 min, 220 °C for 4 min, 220 °C for 4 min, and 220 °C for 4 min. The factors, level tested, and central points of roasting treatment are shown in Table 1. One kilogram of APR sample was placed on a Teflon plate and allowed to run through the SSR conveyor for the roasting process under each of the above conditions. Samples were cooled at room temperature (RT) before sealing in plastic bags to evaluate sensorial properties.

#### 2.2.2. Optimization of Smoking Conditions for APR Processing

Optimal roasting conditions were applied before the smoking treatment. The roasted APR samples were placed on stainless steel plates and arranged in a smoker chamber (Braai Smoker BSTD6; Bradley Technologies Canada Inc., Delta, BC, Canada) for smoking treatment using an oak sawdust puck. The smoking conditions were optimized by using two factors as independent variables (temperature and time) in a central composite design. The 11 combinations of RSM-determined smoking treatments were 60 °C for 10 min, 70 °C for 10 min, 60 °C for 20 min, 70 °C for 20 min, 58 °C for 15 min, 72 °C for 15 min, 65 °C for 8 min, 65 °C for 22 min, 65 °C for 15 min, 65 °C for 15 min, and 65 °C for 15 min. The factors, levels tested, and central points of smoking treatments are shown in Table 2. Smoked APR samples were cooled to RT and packaged in plastic bags before the sensorial properties were evaluated.

### 2.3. Evaluation of Sensorial Properties

The sensorial properties of the samples subjected to the smoke-roasted APR optimization process and the stored final products were evaluated based on their appearance, aroma, taste, texture, and overall acceptance (OA). Approximately 10 g of samples on white dishes, as well as mineral water, a paper cup, stainless spoon, chopsticks, and plain biscuits, were served to 20 selected panelists (25–40 years old). The panelists evaluated the sensory properties using a hedonic scale of 1 to 9, representing dislike extremely (1), dislike very much (2), dislike moderately (3), dislike slightly (4), neither like nor dislike (5, set as threshold), like slightly (6), like moderately (7), like very much (8), and like extremely (9). Each test group was conducted at different times with the same panelists. In total, 200–250 g of sample was warmed and divided into approximately 10 g per serving before evaluating sensorial properties. Samples from the optimization processes were evaluated after microwaving for 30 s at 700 W, and the shelf life test samples (stored final products) were microwaved for 1.5 min at 700 W. Samples were encoded before serving and evaluated randomly by a panelist. The sensorial properties were evaluated at 3 p.m. for a maximum of 2 h in a designated room. Sensorial properties were evaluated with the approval of the institutional review board of Silla University, Busan, Korea.

### 2.4. Evaluation of Chemical Properties

The chemical properties of smoke-roasted APR were evaluated based on the content of volatile basic nitrogen (VBN) and thiobarbituric acid reactive substances (TBARS). Each parameter was measured in triplicate using 5 g of the sample. The VBN content was determined by homogenizing the sample and distilled water (DW, 25 mL) using a homogenizer (WiseTis SHG-15D; SciLab Co. Ltd., Seoul, Korea). The supernatant was collected by centrifugation (4 °C, 3000 rpm for 10 min) and filtration of homogenized samples. Conway micro-diffusion was used to analyze VBN content in the supernatant. 

The TBARS content was measured by homogenizing the sample and 20% trichloroacetic acid in 2 M phosphoric acid (12.5 mL). The volume of the homogenized sample was made up to 25 mL using DW, centrifuged, and filtered to obtain the supernatant. Next, the supernatant (2 mL) was mixed with 0.005 M thiobarbituric acid (TBA, 2 mL) and then incubated in a water bath at 95 °C. Finally, after 30 min of incubation, the absorbance of the mixture was determined at 530 nm using a SPECTROstar Nano microplate reader (BMG Labtech Ltd., Ortenberg, Germany).

The content of Benzo[a]pyrene (B*a*P) was also measured in the final product of smoke-roasted APR to determine its food safety. The measurement was performed using high-performance liquid chromatography (HPLC, Dionex, Sunnyvale, CA, USA) equipped with a fluorescence detector (FLD, Waters 474, Milford, MA, USA) as described in a previous study by Cho et al. [28]. A sample of 10 µL was eluted by 80% acetonitrile in water through a Supelcosil LC-PAH C18 column 5 µm; 4.6 mm i.d. × 250 mm (Supelco Inc., Bellefonte, PA, USA) with a flow rate of 1 mL/min. The detector wavelength was set at 294 nm for excitation and 404 nm for fluorescence. The calculation of B*a*P concentration was performed by comparing it to the standard solution. The standard solution was adjusted at a concentration of 4.158–41.580 µg/kg to make a standard solution curve. The equipment detection limit and assay limit of quantitation were 0.5 and 0.9 µg/kg, respectively. The sample preparation and the measurement of B*a*P were performed by the Korea Analysis Center Co. Ltd. (Busan, Korea).

### 2.5. Evaluation of Microbial Properties

The microbial properties of smoke-roasted APR, including the total bacterial count (TBC), *Escherichia coli*, *Salmonella* spp., and *Staphylococcus aureus* colonies, were evaluated. Analysis was performed in triplicate in Petrifilm plates (3M Korea Ltd., Seoul, Korea). Each sample (10 g) was mixed with sterile 0.9% saline solution (90 mL) in a sterile plastic bag. The mixture was then homogenized using a BagMixer (Interscience Co., Ltd., Osaka, Japan) for 2 min. Thereafter, 1 mL of the homogenous mixture was pipetted out and spread on TBC-, *E. coli*-, *Salmonella* spp-., and *S. aureus-*specific Petrifilm plates, which were then incubated at 35 °C for 2 days. The number of microbes was counted and calculated as log CFU/g, according to the manufacturer’s instructions.

### 2.6. Evaluation of Nutritional Composition

The nutritional composition of raw and smoke-roasted APR samples, comprising 14 mandatory dietary ingredients, including calories, fat, saturated fat, trans fat, cholesterol, sodium, total carbohydrate, dietary fiber, total sugars, protein, vitamin D, calcium, iron, and potassium, was evaluated by the Agricultural Science Research Institute, Chungnam National University, according to the standard methods of the Association of Official Analytical Chemists [29]. Fatty acid and amino acid compositions were measured as described in a previous study [23] using a gas chromatography–mass spectrophotometer (Shimadzu GC—2010 Plus; Shimadzu Co., Kyoto, Japan) and an amino acid analyzer (Hitachi L-8900; Hitachi High-Tech Co., Tokyo, Japan), respectively.

### 2.7. Evaluation of Shelf Life

The shelf life of smoke-roasted APR was evaluated at three different storage temperatures, including 5, 10, and 15 °C, for 30 days. Storage temperatures were set according to the storage temperature of market refrigerators. The evaluation of shelf life was based on OA, VBN, TBARS, and TBC values at 0, 4, 8, 10, 14, 20, and 30 days of storage. Data were analyzed using the Arrhenius equation as described in a previous study [23]. The calculation was performed using the Visual Shelf life Simulator for Foods from the Ministry of Food and Drug Safety (MFDS), Korea (https://www.foodsafetykorea.go.kr, accessed on 12 October 2021). The product rejection value limits of OA, VBN, TBARS, and TBC were 5 score, 35 mg%, 8 mg MDA/kg, and 5 log CFU/g, respectively. The lowest shelf life among the measured parameters was multiplied by a safety factor of 0.82 [30].

### 2.8. Statistical Analysis

Data for optimizing roasting and smoking treatments were analyzed using RSM. Data on drip loss, sensorial, chemical, and microbial properties were analyzed by analysis of variance (ANOVA) followed by Tukey’s *post hoc* multiple comparison tests (*p* < 0.05). All statistical analyses were performed using Minitab ver. 19.0 (Minitab Inc, State College, PA, USA).

## 3. Results and Discussion

### 3.1. Evaluation of Thawing Method of Frozen APR

**Figure 2:**
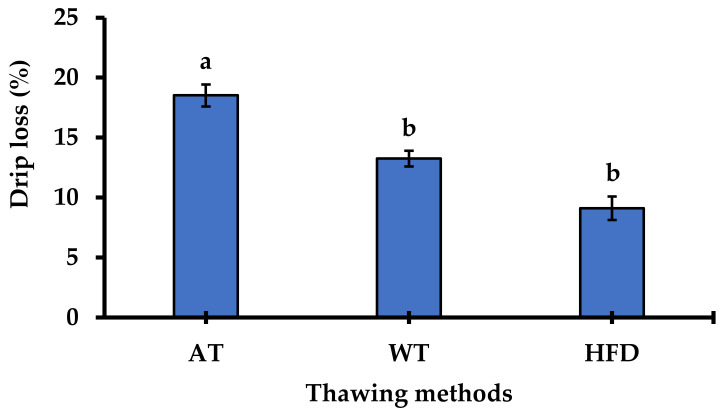
Drip loss (%) of frozen Alaska pollack roe (APR) after thawing using air thawing (AT), water thawing (WT), and high-frequency defrosting (HFD). The data are shown as the mean ± standard error of the mean. Means with different letters differed significantly between thawing methods (Tukey’s test, *n* = 3, *p* < 0.05).

This study used frozen whole APR samples that had not been damaged or dissociated from their roe sac tissue (ovaries). It is important to understand the weight loss of food products because of its economic implications. Weight loss occurs due to drip loss, which includes tissue fluid extrusion and dripping during frozen food thawing. The use of roe in the ovary was intended to prevent high drip loss. In addition, undesirable shapes were avoided after further processing. Dissociated or damaged ovaries potentially increase drip loss due to broken connective tissue. Moreover, roe quality may decrease due to the loss of water-soluble proteins and micronutrients associated with drip loss [31,32]. Therefore, the proper selection of the raw product is critical before further processing. Different thawing methods showed a significant difference in drip loss of frozen APR, as shown in Figure 2 (*p* < 0.05).

Thawing frozen APR using HFD resulted in the lowest drip loss, although it was not significantly different from that of WT. However, WT (75 min) took a longer time than HFD (25 min), while AT (90 min) took the longest time. Thus, HFD could prevent high drip loss and shorten the thawing process. Appropriate methods for achieving minimum drip loss in frozen foods are essential for food processing. Additionally, a quick thawing process is vital to retain raw material quality while minimizing damage to the cell membranes [33,34]. HFD has been shown to lower drip loss during the thawing of fish, squid, and the adductor muscle of the pen-shell (AMPS) compared with that of AT and WT [35,36]. Therefore, HFD was selected as the APR thawing method in the present study to maintain APR quality.

### 3.2. Optimal Roasting and Smoking Conditions of APR

Optimal roasting and smoking conditions were analyzed using the RSM. Several combinations of processing temperature (X_1_) and time (X_2_) were set as independent variables, while sensorial properties were set as dependent variables (Y). The design of the experiment and sensorial property response (appearance, aroma, taste, texture, and OA) are shown in Table 3. 

The sensorial properties were analyzed to obtain the model equation. Initially, response surface regression analysis was performed using a full quadratic model. The models were determined based on the significance of ANOVA in response surface regression. Therefore, only statistically significant (*p* < 0.05) responses and terms were further tested for model determination. The models and their fitness values are listed in Table 4. The full quadratic models were not significant for the interaction term. Thus, the predictive models were re-tested by removing these interactions. All responses showed a significant linear quadratic model, except for the appearance of smoke-roasted APR, indicating that smoking did not result in significant differences in appearance among the various smoking conditions in this study. The roasting treatment of APR showed different responses for all sensory properties. The coefficient of determination (R^2^) indicates the level of model fit to the response data [36]. Texture had the lowest R^2^ value in both roasted and smoke-roasted APRs, implying that different combinations of roasting and smoking temperature and time had less effect on texture than on other sensory properties. Compared with aroma and texture, the appearance, taste, and OA of roasted APR had a greater R^2^, indicating that roasting APR substantially influenced these variables. 

Since appearance did not significantly differ in the smoking treatment, the corresponding predictive model was not built in the optimization process. In contrast to roasting, smoking gave high R^2^ values not only for taste and OA, but also for aroma. The purpose of smoking is to provide a distinctive aroma and taste experience, leading to an increase in the OA score when evaluating sensorial properties [16,37]. The *p*-value for the model’s lack of fit in Table 4 was not significant (*p* > 0.05). Thus, the developed models accurately predicted the optimal temperature and time for both roasting and smoking. 

**Figure 3:**
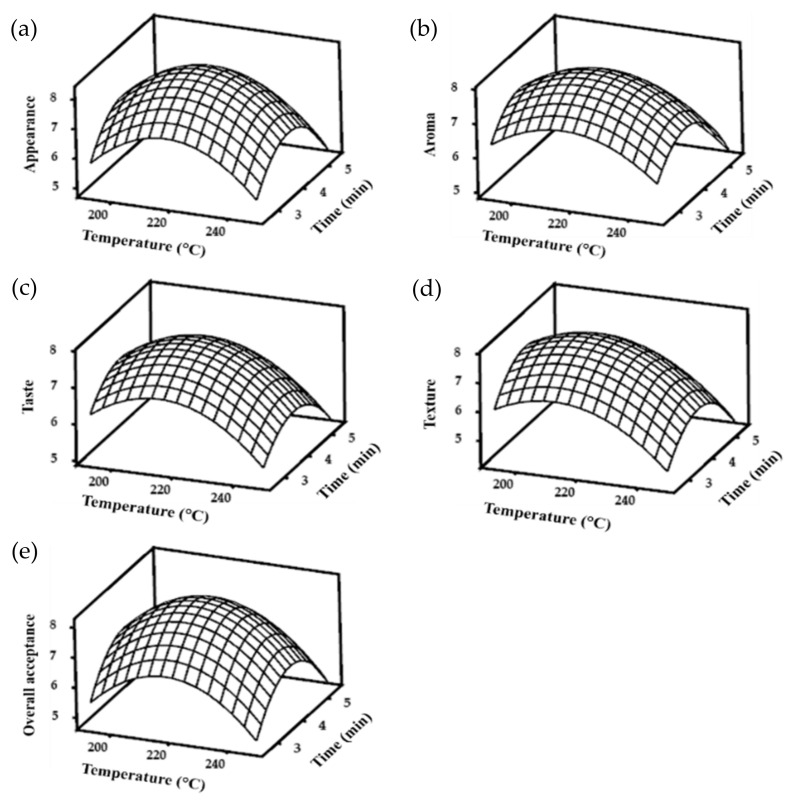
The response wireframe graph for (**a**) appearance, (**b**) aroma, (**c**) taste, (**d**) texture, and (**e**) overall acceptance of roasted Alaska pollack roe at the designated temperature and time.

**Figure 4:**
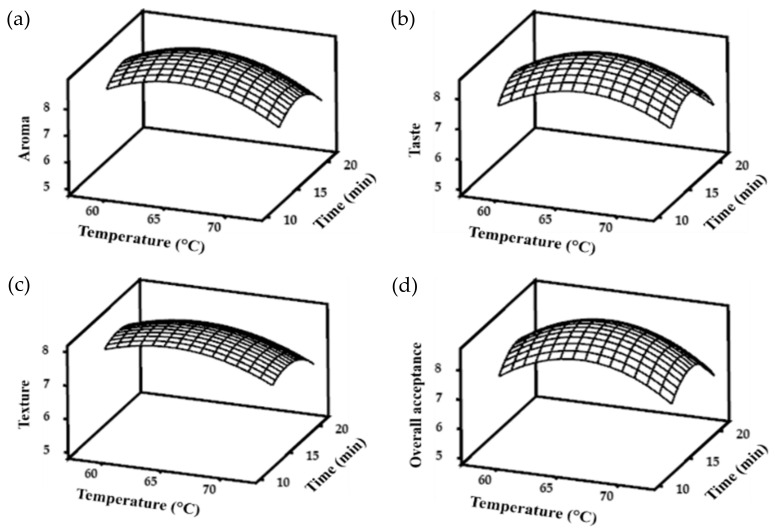
The response wireframe graph for (**a**) aroma, (**b**) taste, (**c**) texture, and (**d**) overall acceptance of smoke-roasted Alaska pollack roe at the designated temperature and time.

The predictive models were used to build the three-dimensional response wireframe graph of sensory properties of roasted and smoke-roasted APR. These graphs show the relationship between the temperature and time settings used for both roasted and smoke-roasted APR. Roasting at shorter time intervals resulted in undercooked APR and low sensorial scores (Figure 3). Undercooked conditions might cause a fishy taste and aroma APR, while overcooking might produce a bitter taste and burnt aroma. Similarly, the smoke-roasted APR developed lower sensorial scores with longer times and temperatures of smoking (Figure 4). This might be due to the product accumulating substances from combustion. In addition, the product could get dryer and its texture might be altered. 

To obtain optimal roasting and smoking conditions, the analysis was performed using the Response Optimizer in the Minitab program for significant model responses. The optimal roasting conditions were 215.8 °C for 3.9 min, rounded to 216 °C for 4 min following the available setting on the SSR. The samples were roasted under optimal conditions for the optimization of smoking conditions. The optimal smoking conditions were obtained at 64.4 °C for 13.8 min, rounded to 64 °C for 14 min.

### 3.3. Nutritional Composition

The nutritional composition of smoke-roasted APR was evaluated and compared with that of seasoned raw APR based on proximate (Table 5), amino acid (Table 6), and fatty acid (Table 7) compositions. The 14 dietary ingredients were evaluated as recommended by the US Food and Drug Administration for nutritional labeling. The results show that seasoned raw and smoke-roasted APR did not contain sugars, trans fat, dietary fiber, or vitamin D. The processing of APR by roasting and smoking increased all measured dietary ingredients due to an increase in dry matter. The roasting treatment decreased the APR moisture as a pre-smoking treatment to prolong shelf life [17]. The highest nutrient content in both seasoned raw and smoke-roasted APR was protein. Consuming 100 g of smoke-roasted APR provides up to 60% of the daily protein needs; the protein content of seasoned raw APR (22%) was higher than that previously reported (19%) [8,9]. The nutritional composition of roe can be affected by roe species and maturity, fish environment, and processing conditions. In addition, different environmental factors impact diet composition and, as a result, influence roe content [10,38]. Park et al. [39] showed that the protein content of APR ranged from 17.27 to 21.79%, with larger APR having higher protein content. The fat content of APR (2.8%) is lower than that of salmon (12.7%), caviar (14.6–15.9%), and mullet (36.8%) [1]. Salmon produces high-fat and -protein roe to provide a consistent supply of nutrients to newborns for extended periods in nutrient-poor environments. However, marine fish roe, such as APR, have little nutrient storage owing to their high-nutrient environment [9].

The development of ready-to-eat APR in the present study resulted in lower sodium levels than processing by salting and fermentation (3.6–7.0%) [13,41]. The sodium content of seasoned raw and smoke-roasted APR was 0.15 and 0.22%, respectively. The seasoned raw APR in this study had a lower sodium level than that reported by Vasconi et al. [9]. This might be due to pre-treatment during seasoning, which involved washing and disinfection treatments. The salt content in smoke-roasted APR contributed 9.8% of the daily value, making smoke-roasted APR a good protein source with a low risk of diseases associated with high salt intake.

The amino acid profile, both essential and non-essential, of smoke-roasted APR showed an increasing trend similar to that of protein content. The essential amino acids of raw and processed APR were lower than those of the non-essential amino acids (Table 6). The total amino acid content of raw APR (20%) was similar to that reported in previous studies [12]. Smoke-roasted APR is rich in leucine, lysine, alanine, aspartic acid, and glutamic acid. These amino acid contents are commonly high in fish roes, as reported by Mol et al. [1]. The leucine content of seasoned raw APR (1.9%) was higher than that of various caviars (0.28–0.37%), red salmon roe (0.46%), and mullet roe (0.33%), but the lysine content was 41–75% lower than that of other roes. The aspartic acid (1.6%) and glutamic acid (2.9%) contents in seasoned raw APR were lower than those in other fish roes (2.9–3.3% and 4.5–4.9%, respectively). The differences in these contents are related to the species and age of the fish and roe, and the fish habitat [1], and are increased by roasting and smoking treatments due to the increase in dry matter. The high aspartic and glutamic acid content in smoke-roasted APR might contribute to the umami and sweet tastes [41]. Furthermore, Lee [13] found that processed APR with a high amino nitrogen content also had a high sensory attribute score.

The 4.3% fat content of smoke-roasted APR consisted of fats good for human consumption. As shown in Table 7, almost 50% of the fat consisted of polyunsaturated fatty acids (PUFAs). EPA and DHA are the major fatty acids in APR, thereby increasing PUFA content. Other major fatty acids were palmitic and oleic acids, such as saturated fatty acids (SFA) and monounsaturated fatty acids (MUFAs), respectively. Comparable results were reported by Mol and Turan [1], who observed that fish roe fatty acids are highly abundant in palmitic and oleic acids, EPA, and DHA. The total ꞷ-3 fatty acids of raw and processed APR were 44.32–44.34%, while total ꞷ-6 was 2.01–2.17%. The high total amount of ꞷ-3 fatty acids resulted in a low ꞷ-6 and ꞷ-3 ratio (0.045–0.049). These conditions are favorable for the human diet because higher ꞷ-6 and ꞷ-3 ratios have the potential to increase the risk of cardiovascular, inflammatory, and autoimmune diseases [42]. Therefore, healthy diets are recommended to have a ꞷ-6 to ꞷ-3 ratio of 1–4 or below [43]. The fatty acid composition showed similar results after roasting and smoking treatments of seasoned raw APR. 

### 3.4. Quality of Smoke-Roasted APR during Storage

The quality of smoke-roasted APR was indicated by OA, VBN, TBARS, and TBC. The changes in these indicators are shown in Table 8. The quality of the product decreased significantly with increasing storage temperature and time (*p* < 0.05). Sensory and microbial changes indicate product quality, which may affect the shelf life of processed fish [44]. OA decreased significantly on day 14 of storage at temperatures of 5 and 10 °C, and decreased more rapidly on day 8 at a temperature of 15 °C. (*p* < 0.05). These results show that storage temperatures less than 10 °C maintained better sensorial scores. The chemical and microbial properties of fish products are related to their sensory properties. Furthermore, during the storage period, microbial activity contributes to an unpleasant taste and aroma of the products [45,46]. The OA score represents sensory evaluation performed using a 9-point hedonic scale. The evaluation criteria were determined such that the initial quality level was 9 points and the quality limit was 5 points. However, the smoke-roasted APR showed an OA score of more than 5 during 30 days of storage, which is considered acceptable.

The VBN value of smoke-roasted APR increased significantly, compared with that of seasoned raw APR, from 21.80 to 25.86 mg% (*p* < 0.05), indicating that the increasing VBN value was related to the roasting and smoking treatments. Thermal processing breaks down the protein in foods and increases its digestibility [47]. Since protein breakdown is associated with the VBN value [48], the high temperature during roasting and smoking could increase the VBN values in processed APR. The breakdown of proteins can also be caused by microbial and enzymatic activities [49]. The presence of microbial activity might explain the increase in VBN values during storage in the present study. Table 8 shows that the TBC increased during storage and is positively correlated with VBN values (*r* = 0.745 by Pearson’s correlation). Jeong et al. [50] reported that the VBN values of various grades of raw APR ranged from 15.7 to 28.2 mg%, consistent with the VBN values of APR before processing in the current study. The VBN values tended to increase rapidly at higher storage temperatures. After 30 days of storage, the VBN values of smoke-roasted APR refrigerated at 5 °C increased significantly, but increased more rapidly at 10 and 15 °C after 20 days (*p* < 0.05). The assessment of spoilage based on the VBN value in fish is divided into several categories: perfect (5–10 mg%), good (15–25 mg%), and spoiled (>40 mg%) [50]. Considering the aforementioned categories, only the sample on day 30 at a storage temperature of 15 °C had a VBN value indicating spoilage. Based on these categories, the shelf life of smoke-roasted APR was determined to have a quality limit of 40 mg%.

The TBARS value reflects the amount of malondialdehyde (MDA) produced by lipid oxidation; MDA generates a rancid flavor and is associated with the spoilage of meat and fish products [51,52]. The TBARS value of seasoned raw APR was 0.36 mg MDA/kg, which slightly increased (14%) after smoke-roasting treatment. Food processing time and temperature influences lipid oxidation [53,54]. The use of SSR in the current study facilitated the suppression of APR oxidation, resulting in a negligible increase in TBARS. SSR facilitates faster processing in oxygen-deficient environments, thus minimizing oxidation in foods [55]. TBARS values increased gradually during storage, significantly increasing after 14 days at all storage temperatures (*p* < 0.05). Yildiz [56] categorized TBARS values in seafood into three categories: perfect (<3 mg MDA/kg), good (3–5 mg MDA/kg), and consumable limit (7–8 mg MDA/kg). TBARS values in the present study were low and thus categorized as perfect.

Smoking, charcoal grilling, and roasting are known as the potential sources of B*a*P. B*a*P is one of the carcinogenic polycyclic aromatic hydrocarbons (PAHs) formed by the combustion of wood in smoking process [57]. In this study, B*a*P content was not detected in the smoke-roasted APR. Further research is needed to evaluate the hazardous substances, such as acrylamide, heavy metals, and other PAHs contents, in APR treated by this method. Based on the European Union (EU) regulation, the maximum level of B*a*P in smoked foods is limited to 5.0 µg/kg [58]. As a result, the smoke-roasted APR in this study was considered safe.

Changes in microbial conditions may determine the safety of the food. In the present study, pathogenic microbes, including *E. coli, Salmonella* spp. and *S. aureus,* were not detected during storage at all temperatures. This indicates that raw material processing was performed hygienically. The microbial (TBC) change in smoke-roasted APR during 30 days of storage is shown in Table 8. The TBC of seasoned raw APR significantly decreased (38.6%) after roasting and smoking treatments (*p* < 0.05), but increased with storage time. The highest TBC value was 4.97 log CFU/g, contained in the sample at 15 °C after storage for 30 days. Vacuum packing, pasteurization, and low-temperature storage are some of the methods for reducing microbial activity [17]. Microbial activity is slowed at lower temperatures; hence, temperature reduction reduces the development of spoilage-causing microbes [59]. Unpasteurized smoke-roasted APR contained a TBC of more than 5 log CFU/g after 7 days of storage (data not shown). Farag et al. [2] suggested that the pasteurization temperature for fish roe is between 50 and 70 °C due to its thermal sensitivity. Fish roe preserves its physical appearance within this temperature range, with no protein coagulation affecting protein quality. The smoke-roasted APR samples in the present study were packed under vacuum-pasteurized conditions. Therefore, the TBC values of all samples until 30 days of storage were less than 5 log CFU/g, which is considered safe for human consumption [60]. Thus, the evaluation criteria for shelf life were determined at a TBC quality limit of 5 log CFU/g.

The shelf life of the smoke-roasted APR was estimated according to the changes in the quality indicators in Table 8. As a result, the shelf life of smoke-roasted APR based on OA, VBN, TBARS, and TBC was 41.9, 30.3, 86.0, and 55.8 days. In addition, the shortest shelf life was selected from the calculated quality indicators to determine product shelf life. Accordingly, 30.3 days was selected as the shortest shelf life, which was multiplied by 0.82 as the safety factor. This safety factor considers temperature changes during distribution in the market [30]. Therefore, the shelf life of the product was 24 days. Studies on changes in product quality in frozen storage could be considered for further research in order to obtain a longer shelf life.

## 4. Conclusions

This study showed that the optimum processing conditions determined using RSM resulted in good sensorial properties. The combination of superheated steam roasting and smoking in APR improved its nutritional content, OA, and microbial properties. Additionally, the chemical properties (VBN and TBARS) slightly increased, but were at an acceptable level. Furthermore, the chemical and microbial properties gradually increased during storage for 30 days at refrigeration temperature, resulting in a decrease in OA. Therefore, this product is safe and has an acceptable quality for up to 24 days when stored at refrigerator temperature. These results suggest that combined superheated steam roasting and smoking have potential in the development of ready-to-eat APRs.

## Figures and Tables

**Table 1:** Factors and their levels in the three points of central composite design for optimizing the SSR conditions of APR.

Factor	Symbol	Level				
Temperature (°C)	X_1_	191	200	220	240	248
Time (min)	X_2_	2.5	3	4	5	5.5

**Table 2:** Factors and their levels in the three points of central composite design for optimizing the smoking conditions of roasted APR.

Factor	Symbol	Level				
Temperature (°C)	X_1_	58	60	65	70	72
Time (min)	X_2_	8	10	15	20	22

**Table 3:** Appearance, aroma, taste, texture, and overall acceptance (OA) of roasted and smoke-roasted Alaska pollack roe (APR) in a central composite design.

Treatment	Temp (°C)	Time (min)	Appearance	Aroma	Taste	Texture	OA
Roasted APR	200	3.0	7.00	7.14	7.00	6.86	6.71
	240	3.0	6.88	7.14	6.43	5.43	6.43
	200	5.0	6.79	6.86	6.71	6.71	6.58
	240	5.0	6.43	6.57	6.21	5.57	6.36
	192	4.0	7.13	7.12	7.07	7.21	7.03
	248	4.0	6.58	6.43	6.14	6.29	6.43
	220	2.5	6.97	7.00	6.86	6.86	6.74
	220	5.5	6.56	5.86	6.57	6.14	6.57
	220	4.0	8.27	7.71	7.89	7.43	8.14
	220	4.0	8.14	8.17	7.54	7.57	8.11
	220	4.0	8.31	7.68	7.43	7.84	8.03
Smoke-roasted APR	60	10.0	7.43	8.43	7.80	7.55	7.63
	70	10.0	7.43	8.23	7.93	7.48	7.70
	60	20.0	7.80	8.35	7.28	7.18	7.55
	70	20.0	7.93	7.60	7.43	6.95	7.35
	58	15.0	8.05	8.15	7.63	7.68	7.68
	72	15.0	7.80	7.65	7.43	7.06	7.43
	65	8.0	8.30	8.33	7.65	7.68	7.98
	65	22.0	7.68	7.88	7.33	7.30	7.52
	65	15.0	8.08	8.73	8.43	7.55	8.51
	65	15.0	7.93	8.80	8.50	7.85	8.38
	65	15.0	8.13	9.00	8.30	8.03	8.66

**Table 4:** The response surface model of appearance, aroma, taste, texture, and overall acceptance (OA) of roasted and smoke-roasted Alaska pollack roe (APR) with different temperatures (X_1_) and times (X_2_).

Treatment	Response	Predictive Model	*R*^2^ (%)	Lack of Fit (*p*-Value)
Roasted APR	Appearance	−89.18 + 0.80X_1_ + 5.27X_2_ − 0.0018X_1_^2^ − 0.68X_2_^2^	98.70	0.43
	Aroma	−57.30 + 0.53X_1_ + 4.37X_2_ − 0.0012X_1_^2^ − 0.58X_2_^2^	89.74	0.58
	Taste	−61.34 + 0.58X_1_ + 3.28X_2_ − 0.0014X_1_^2^ − 0.42X_2_^2^	95.17	0.94
	Texture	−66.00 + 0.61X_1_ + 4.66X_2_ − 0.0014X_1_^2^ − 0.60X_2_^2^	83.78	0.17
	OA	−92.40 + 0.82X_1_ + 5.42X_2_ − 0.0019X_1_^2^ − 0.68X_2_^2^	97.26	0.09
Smoke-roasted APR	Aroma	−64.80 + 2.23X_1_ + 0.37X_2_ − 0.017X_1_^2^ − 0.013X_2_^2^	91.36	0.40
	Taste	−65.90 + 2.18X_1_ + 0.49X_2_ − 0.017X_1_^2^ − 0.018X_2_^2^	93.65	0.30
	Texture	−35.90 + 1.32X_1_ + 0.20X_2_ − 0.010X_1_^2^ − 0.008X_2_^2^	80.67	0.79
	OA	−80.64 + 2.65X_1_ + 0.47X_2_ − 0.021X_1_^2^ − 0.016X_2_^2^	96.17	0.73

**Table 5:** Nutritional composition of seasoned raw and smoke-roasted APR (per 100 g).

Nutrient	Seasoned Raw APR	Smoke-Roasted APR	% Daily Value	Daily Value *
Carbohydrate (g)	4.5	8.1	2.9	275
Sugars (g)	0	0	0.0	50
Crude protein (g)	22	30	60.0	50
Fat (g)	2.8	4.3	5.5	78
Saturated fat (g)	0.5	0.8	4.0	20
Trans fat (g)	0	0	0.0	2
Cholesterol (mg)	338.7	516.8	172.3	300
Sodium (mg)	148.8	224.6	9.8	2300
Dietary fiber (g)	0	0	0.0	28
Vitamin D (μg)	0	0	0.0	20
Calcium (mg)	9.9	12.9	1.0	1300
Iron (mg)	0.8	1.1	6.1	18
Potassium (mg)	149.4	156.1	3.3	4700
Calorie (kcal)	131.2	191.1	-	-

* According to the nutritional labeling requirements of the US FDA [40]. The % daily value was calculated based on the nutritional content of smoke-roasted Alaska pollack roe (APR).

**Table 6:** Amino acid profile of seasoned raw and smoke-roasted Alaska pollack roe (APR) (g/100 g).

Amino Acid	Seasoned Raw APR	Smoke-Roasted APR
Histidine	0.40	0.59
Isoleucine	1.18	1.65
Methionine	0.57	0.78
Leucine	1.94	2.80
Lysine	1.55	2.20
Phenylalanine	0.86	1.22
Threonine	0.93	1.39
Tryptophan	0.24	0.34
Valine	1.31	1.82
Alanine	1.60	2.29
Arginine	1.03	1.50
Aspartic acid	1.65	2.40
Cystine	0.36	0.54
Glutamic acid	2.91	4.14
Glycine	0.66	0.97
Proline	1.18	1.72
Tyrosine	0.65	1.06
Serine	1.04	1.60
Total essential	8.98	12.79
Total non-essential	11.08	16.22

**Table 7:** Lipid profile of seasoned raw and smoke-roasted Alaska pollack roe (APR) (% of total lipids).

Fatty Acid	Seasoned Raw APR	Smoke-Roasted APR
Capric acid	0.01	0.01
Lauric acid	0.01	0.01
Myristic acid	3.18	3.63
Pentadecanoic acid	0.30	0.33
Palmitic acid	22.12	21.53
Magaric acid	0.10	0.12
Stearic acid	1.68	1.85
Arachidic acid	0.04	0.04
Lignoceric acid	1.67	1.30
Heneicosylic acid	0.02	0.06
Myristoleic acid	0.06	0.06
Pentadecenoic acid	0.05	0.06
Palmitoleic acid	5.33	5.94
Oleic acid	16.54	16.19
Magaoleic acid	0.15	0.14
Eicosenoic acid	2.05	1.97
Erucic acid	0.34	0.28
Linoleic acid	0.97	0.86
γ-Linolenic acid	0.07	0.07
Eicosadienoic acid	0.20	0.19
Dihomoδ-Linoleic acid	0.06	0.05
Arachidonic acid	0.71	1.00
Linolenic acid	0.28	0.42
Eicosatrienoic acid	0.06	0.08
EPA	20.40	19.86
DHA	23.60	23.96
Total SFA	29.13	28.88
Total MUFA	24.52	24.64
Total PUFA	46.35	46.49
Total ꞷ-3	44.34	44.32
Total ꞷ-6	2.01	2.17
ꞷ-6/ꞷ-3	0.045	0.049

Notes: SFA, saturated fatty acid; MUFA, monounsaturated fatty acid; PUFA, polyunsaturated fatty acid; EPA, eicosapentaenoic acid; DHA, docosahexaenoic acid.

**Table 8:** The changes in overall acceptance (OA), volatile basic nitrogen (VBN), thiobarbituric acid reactive substances (TBARS), and total bacterial count (TBC) in seasoned raw Alaska pollack roe (APR) and smoke-roasted APR during storage at three different refrigerator temperatures.

Storage		Quality Indicator			
Temp.	Day	OA (Score)	VBN (mg %)	TBARS (mg MDA/kg)	TBC (Log CFU/g)
5 °C	0	8.89 ± 0.11 ^a^	25.86 ± 0.31 ^a,^*	0.41 ± 0.02 ^a^	1.88 ± 0.05 ^a,^*
	4	8.67 ± 0.17 ^a^	25.67 ± 1.99 ^a^	0.41 ± 0.02 ^a^	1.94 ± 0.06 ^a^
	8	8.44 ± 0.18 ^a,b,c^	26.83 ± 2.37 ^a,b^	0.42 ± 0.03 ^a^	1.81 ± 0.25 ^a^
	14	7.67 ± 0.17 ^c,d,e^	28.00 ± 1.13 ^a,b,c^	0.44 ± 0.02 ^a^	2.01 ± 0.31 ^a^
	20	6.78 ± 0.15 ^f,g^	36.05 ± 1.41 ^a,b,c,d^	0.70 ± 0.04 ^b,c,d^	2.24 ± 0.17 ^a^
	30	6.67 ± 0.17 ^f,g,h^	38.38 ± 2.73 ^b,c,d^	0.80 ± 0.02 ^d,e^	2.35 ± 0.17 ^a^
10 °C	0	8.89 ± 0.11 ^a^	25.86 ± 0.31 ^a^	0.41 ± 0.02 ^a^	1.88 ± 0.05 ^a^
	4	8.56 ± 0.18 ^a,b^	31.62 ± 0.31 ^a,b,c,d^	0.44 ± 0.03 ^a^	1.97 ± 0.20 ^a^
	8	8.22 ± 0.15 ^a,b,c^	33.37 ± 1.91 ^a,b,c,d^	0.45 ± 0.02 ^a^	1.97 ± 0.20 ^a^
	14	7.33 ± 0.24 ^d,e,f^	33.25 ± 1.21 ^a,b,c,d^	0.48 ± 0.02 ^a,b,c^	2.34 ± 0.39 ^a^
	20	6.44 ± 0.18 ^g,h^	38.62 ± 5.06 ^c,d^	0.71 ± 0.03 ^c,d^	2.45 ± 0.15 ^a^
	30	5.88 ± 0.20 ^h,i^	39.90 ± 2.83 ^d^	0.98 ± 0.02 ^e,f^	3.25 ± 0.40 ^a^
15 °C	0	8.89 ± 0.11 ^a^	25.86 ± 0.31 ^a^	0.41 ± 0.02 ^a^	1.88 ± 0.05 ^a^
	4	8.33 ± 0.17 ^a,b,c^	31.03 ± 0.62 ^a,b,c,d^	0.51 ± 0.01 ^a,b,c^	1.89 ± 0.05 ^a^
	8	7.78 ± 0.15 ^b,c,d^	33.13 ± 2.44 ^a,b,c,d^	0.46 ± 0.02 ^a,b^	1.89 ± 0.05 ^a^
	14	6.89 ± 0.20 ^e,f,g^	33.83 ± 0.51 ^a,b,c,d^	0.51 ± 0.11 ^a,b,c^	2.27 ± 0.27 ^a^
	20	6.33 ± 0.17 ^g,h,i^	39.78 ± 4.34 ^d^	0.84 ± 0.07 ^d,e,f^	3.32 ± 1.03 ^a,b^
	30	5.56 ± 0.24 ^i^	41.65 ± 1.76 ^d^	1.08 ± 0.12 ^f^	4.97 ± 0.28 ^b^
Seasoned RAW APR		-	21.80 ± 0.56 *	0.36 ± 0.04	3.06 ± 0.11 *

Notes: Smoke-roasted APR samples were stored at 5, 10, and 15 °C. Values are expressed as mean ± standard error of the mean. The means of each attribute with different letters (^a–i^) were significantly different according to Tukey’s test (*p* < 0.05). Asterisks (*) indicate significant differences between seasoned raw APR and smoke-roasted APR on day zero.

## Data Availability

Data supporting reported results are available upon request.
